# In Situ Ring-Opening Polymerization of *L*-lactide on the Surface of Pristine and Aminated Silica: Synthesis and Metal Ions Extraction

**DOI:** 10.3390/polym14224995

**Published:** 2022-11-18

**Authors:** Liliia M. Polishchuk, Roman B. Kozakevych, Andrii P. Kusyak, Valentin A. Tertykh, Oleg Tkachenko, Maria Strømme, Tetyana M. Budnyak

**Affiliations:** 1 Chuiko Institute of Surface Chemistry, 17 General Naumov Street, 03164 Kyiv, Ukraine; 2 The Ångström Laboratory, Division of Nanotechnology and Functional Materials, Department of Materials Science and Engineering, Uppsala University, Lägerhyddsvägen 1, 751 03 Uppsala, Sweden

**Keywords:** *L*-lactide, polylactic acid, ring-opening polymerization, polylactide-silica composites, toxic metal ions, adsorption

## Abstract

The development of functional materials from food waste sources and minerals is currently of high importance. In the present work, polylactic acid (PLA)/silica composites were prepared by *in situ* ring-opening polymerizations of *L*-lactide onto the surface of pristine (Silochrom) and amine-functionalized (Silochrom-NH_2_) silica. The characteristics of the ring-opening polymerization onto the surface of modified and unmodified silica were identified and discussed. Fourier transform infrared spectroscopy was used to confirm the polymerization of lactide onto the silica surface, and thermogravimetric analysis determined that PLA constituted 5.9% and 7.5% of the composite mass for Silochrom/PLA and Silochrom-NH_2_/PLA, respectively. The sorption properties of the composites with respect to Pb(II), Co(II), and Cu(II) ions were investigated, and the effect of contact time, initial metal ion concentration, and initial pH were evaluated. Silochrom-NH_2_/PLA composites were found to have a higher adsorption capacity than Silochrom/PLA for all chosen ions, with the highest adsorption value occurring for Pb^2+^ at 1.5 mmol/g (90% removal efficiency). The composites showed the highest performance in the neutral or near-neutral pH (created by distilled water or buffer pH 6.86) during the first 15 min of phase contact. The equilibrium characteristics of adsorption were found to follow the Langmuir isotherm model rather than the Freundlich and Temkin models. Perspective applications for these PLA/silicas include remediation of industrial wastewater or leaching solutions from spent lead-acid and Li-ion batteries.

## 1. Introduction

**Figure 1:**
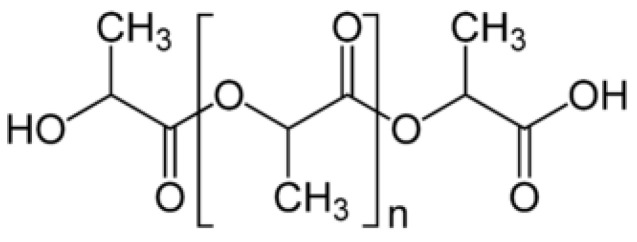
The structural formula of Polylactic acid (PLA).

Water pollution caused by toxic heavy metal ions has become a serious global environmental problem. Due to their resistance, inseparability, and toxicity, heavy metal ions pose a serious threat to water resources and overall human health, even at low concentrations. Of all the remediation techniques, adsorption is one of the most effective and promising methods for metal ion removal. Specifically, many sorption processes are low cost, require minimum maintenance, show high efficiency, and ease of operation. However, some of the key challenges in this field are the low adsorption capacity, relatively weak interactions with metallic ions, and difficulties surrounding the separation and regeneration of absorbent from water. Organic-inorganic composites [1,2] are promising materials for the extraction of toxic compounds from water. These heterophase materials consist of two or more components of different chemical nature with different physicochemical properties [3,4]. One class of these materials is functionalized hybrid polymeric composites, which have been regarded as one of the most effective adsorbents. These kinds of materials often present the best properties of each of their components in a synergic way and have been shown to have excellent physical, chemical, and mechanical properties. Recently, sustainability has become an important aspect of materials production as a way of reducing the impact on the environment. As a result, more attention has been paid to biopolymeric products based on biodegradable materials [5]. Polylactic acid (PLA) is a biodegradable aliphatic polyester derived from 100% renewable resources, such as corn and sugar beets [6]. Moreover, it has unique physical properties that make it useful in diverse applications, including medical applications, tissue engineering, adsorbent material, and drug delivery systems. Polylactic acid is a hydrophobic polymer that belongs to the class of biomaterials commonly referred to as poly-α-hydroxy acids, poly-α-esters, or aliphatic polyesters (Figure 1).

PLA synthesis proceeds either through step-growth polymerization or ring-opening polymerization (ROP). Step growth polymerization simply takes advantage of the reactivity of the two functional groups. Specifically, the polycondensation of hydroxyl and carboxyl moieties leads to the formation of the ester bonds that constitute the polymer backbone [7]. At an industrial scale, ROP is the most popular process because of the mild process conditions, short residence times, absence of side products, and the ability to achieve high molecular weights [8]. The most widely used catalyst is 2-ethylhexanoic tin(II) salt (also referred to as stannous octoate [Sn(Oct)_2_]), which has been approved by the United States Food and Drug Administration (FDA) and is usually employed along with alcohol as co-catalyst. PLA is soluble in dioxane, acetonitrile, chloroform, methylene chloride, 1,1,2-trichloroethane, and dichloroacetic acid, while it is only partially soluble in ethylbenzene, toluene, acetone, and tetrahydrofuran when heated to boiling temperature. PLA is not soluble in water, alcohols, or linear hydrocarbons.

The physical properties of the polymer composites can be altered in hybrid systems by the inclusion of mineral-based nanoparticles. Among the numerous inorganic/organic hybrid materials, silica-polymer hybrid materials are ideal candidates. Silica is well-known as a perspective carrier due to its rigid structure, characterized by thermal stability, high specific surface area, low cost, and applicability at an industrial scale [9,10,11,12,13]. The chemical properties of the silica surface are largely determined by the various silanol and siloxane groups that are present. The hydroxyl groups on the surface of silica particles can be functionalized by silane reagents or polymers. The most common way to activate the silica surface is via amination with a 3-aminopropyltriethoxysilane precursor [14]. Recently, attempts have been made to polymerize L-lactide onto the surface of hydroxyapatite nano-crystals [15] and cellulose nanofibrils [16]. Alba et al. successfully conducted direct ring-opening of lactide with amines, confirming the possibility of lactide modification by variable amines [17]. However, the ring-opening polymerization on the pristine and aminated silica surface is still a challenge.

Therefore, more attention is needed to be focused on polylactide and silica or aminated silica composites, which can have a wide variety of potential applications. This work aims to develop synthesis methods and to study the properties of composites made with PLA and silochrom and aminopropyl-silochrom [18]. The preparation of composite sorbents was carried out by polymerization of *L*-lactide with the opening of its ring on the surface layer of silica. Sorption studies of the composites were carried out with respect to Pb(II), Co(II), and Cu(II) ions, as these are the most abundant components in leaching solutions from spent lead-acid and Li-ion batteries [10,19,20].

## 2. Materials and Methods

### 2.1. Materials

**Figure 2:**
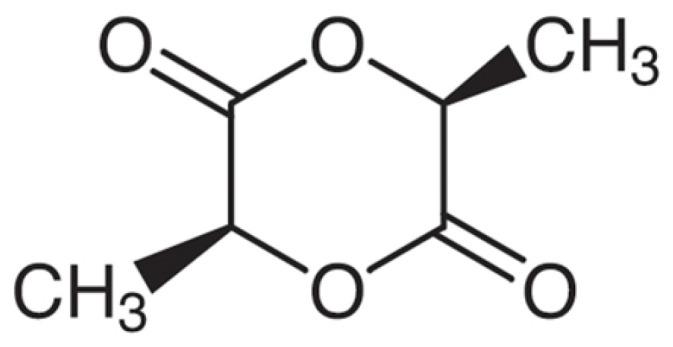
Structural formula of *L*-lactide.

Monomer *L*-lactide (3S,6S)-3,6-dimethyl-1,4-dioxane-2,5-dione (Figure 2) was used as received from Sigma Aldrich (99 wt.%). Stannous octoate (assay ≥ 99%) was purchased from Sigma Aldrich. Porous silica spheres, Silochrom, with a specific surface area of 143 m^2^/g, total pore volume of 0.95 cm^3^/g, and mean pore diameter of 17 nm, were produced via spray-drying technique from highly dispersed non-porous silica particles and were used throughout this study.

### 2.2. Amine-Functionalized Porous Silica Samples (Silochrom-NH_2_) 

Modified silica particles were prepared via the treatment of the pristine silica with 3-aminopropyltriethoxysilane (APTES, 99%, Fluka). Specifically, pristine silica was dehydrated at 200 °C for 2 h and then refluxed for 2 h at 60 °C with APTES in toluene (99.5%, Aldrich) at the ratio 1 g SiO_2_/0.2 g APTES/10 mL toluene. The obtained product was filtered and sequentially washed with toluene and acetone (99.5%, Aldrich), from which the particles were dried at 80 °C for 2 h in air. The content of the chemisorbed amino groups was determined using the spectrophotometric method (spectrophotometer SF-46, absorption wavelength 400 nm) after the reaction of the surface NH_2_-groups with the salicylic aldehyde. The amino group content was calculated as 0.25 mmol per 1 g of silica.

### 2.3. Synthesis of Silochrom/PLA and Silochrom-NH_2_/PLA Composites 

A 250 mL three-neck round-bottom flask equipped with a magnetic stirrer, thermometer, and condenser was used as a polymerization reaction vessel. For the reaction, 5 g of Silochrom or Silochrom-NH_2_, and the appropriate portion of lactide, dissolved in ethanol were introduced into the flask, and the system was heated to 45 °C, under stirring. After 1.5 h, a 2 wt% stannous octoate catalyst was added. The polymerization reaction was carried out in an oil bath at a constant temperature of 100 °C for 2 h [21,22,23]. The synthesis lasted 2 h. After completion of the synthesis, the resulting sorbent was washed several times with ethanol and water, and then it was dried at 100 °C.

### 2.4. Methods

Surface area and porosity were determined from nitrogen adsorption-desorption isotherms obtained at −196 °C using a Micromeritics ASAP-2000 analyzer (USA). In all cases, 50–70 mg of material was degassed at 60 °C for 4 h under a vacuum (residual pressure <10^−5^ Torr) before nitrogen adsorption. The specific surface area of adsorbents was determined by Brunauer–Emmett–Teller (BET) measurements [24] and the pore size distribution by Barrett-Joyner-Halenda (BJH) method [25].

Fourier transform Infrared (FTIR) spectra were collected using a Thermo Nicolet Nexus 450 from 4000 to 500 cm^−1^ and resolution of 4 cm^−1^, using KBr pellets in weight ratio sample: KBr as 1:20.

X-ray photoelectron spectroscopy (XPS) was recorded using PHI Quantera II Scanning XPS Microprobe (Physical Electronics, Chanhassen, MN, USA) to confirm the covalent bonding of L-lactide onto the pristine and aminated silica. The pressure in the chamber was approximately 10^−9^ Pa. An X-ray source of monochromatized Al Kα operating at 100 W was used for the measurements. The passing energy of the hemispheric analyzer was set at 50 eV for the high-resolution spectra. The surface charge compensation was performed by an ion gun. The results were analyzed with the MultiPak software (Physical Electronics). The spectra binding energy scale was correlated, taking as reference the C1s peak at 284.8 eV.

Differential thermal analysis (DTA) and thermogravimetry (TG) were performed using a Derivatograph Q-1500 D (MOM, Mátészalka, Hungary). The average sample mass was 250 mg. The heating rate was 5 °C min^−1^. The thermoanalytical curves were recorded up to a temperature of 1000 °C in the air atmosphere.

The sorption properties of the silochrom-polylactide (silochrom/PLA) and aminosilochrom-polylactide (silochrom-NH_2_/PLA) composites were studied in the static mode with periodic mixing. For this study, 0.1 g of adsorbent per 25 mL was used to isolate the salts of the formed cations: Pb(NO_3_)_2_, CoCl_2,_ and CuSO_4_·5H_2_O, fixing the concentration of cations by the atomic absorption method [26]. Standard buffer solutions (DSTU 8.135: 2009, manufactured by OJSC Kyiv RIAP Plant) were used to create the appropriate pH of the medium.

The removal efficiency (*R*, %) was calculated by the formula:

*R* = (*m*_ads_/*m*_o_) 100% = (*m*_o_ − *m*)/*m*_o_ 100%,

where *m*_o_ is the mass of the metal in the initial solution, *m*_ads_ is the mass of the adsorbed metal, and *m* is the mass of the metal in the equilibrium solution after adsorption, which was calculated as *m* = *C*·*V*, where *C* is the equilibrium metal concentration, and *V* is the volume equilibrium solution.

The equilibrium concentrations of the metals were determined by atomic absorption spectroscopy using a flame atomic absorption spectrophotometer “Saturn” (Kyiv, Ukraine) in an air-propane-butane flame mixture. The characteristic wavelengths for measurements were used: 324.7 nm for Cu, 228.8 nm for Pb, and 248.3 nm for Co. The calculations of the equilibrium concentrations in the solution were made by comparing the intensities of their lines in the spectrum with the intensity of the lines of standard solutions.

## 3. Results and Discussion

### 3.1. Preparation of Polylactic Acid/Silica Composites

Lactide, a cyclic diester of lactic acid, is the monomer used to synthesize PLA (polylactic acid) by ring-opening polymerization (ROP). Lactide is a natural and renewable compound produced from lactic acid (2-hydroxypropanoic acid), which is obtained by the fermentation of sucrose or glucose. Stannous octoate was used as a catalyst for the polymerization of *L*-lactide.

**Scheme 1:**
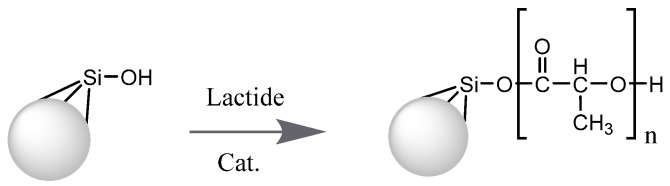
Synthesis silochrom/PLA composite with catalyst.

**Scheme 2:**
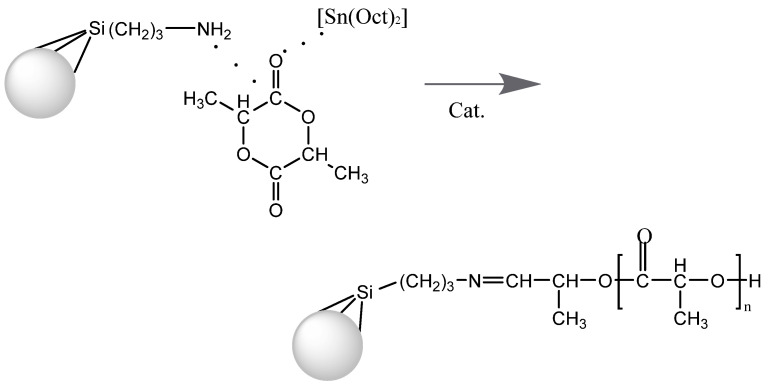
Synthesis of silochrom-NH_2_/PLA composite with catalyst.

Two PLA/silica composites were synthesized via ring-opening polymerization of *L*-lactide onto the surface of porous pristine silica, Silochrom, and amine-functionalized silica, Silochrom-NH_2_, in the presence of stannous octoate catalyst [21,22,23]. Polymerization of L-lactide onto the silica surface is schematically presented in Scheme 1 and Scheme 2 below.

**Figure 3:**
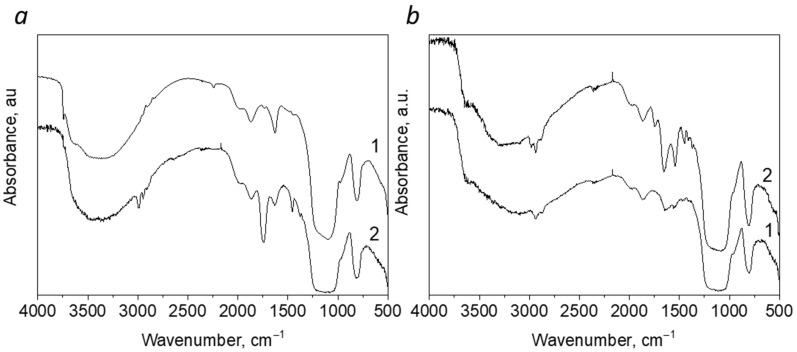
IR spectra of (**a**): Silochrom (1), composite Silochrom/PLA (2); (**b**): Silochrom-NH_2_ (1), composite Silochrom-NH_2_/PLA (2).

The IR spectra of the Silochrom and Silochrom-NH_2_ particles were compared to the spectra of the silica/PLA composites (Figure 3a,b) to confirm the polymerization of L-lactide onto the surface of the silica.

In the spectrum of the starting Silochrom (Figure 3a), the bands at 1090 and 800 cm^−1^ can be attributed to the vibrational modes in the Si-O-Si bond of the silica. Following the polymerization of lactide, an intense absorption band in the C-H stretching region (2950–2900 cm^−1^) and the absorption band at 1740 cm^−1^, attributed to the valence vibrations of the carbonyl group (C=O), appear. At the same time, the band intensity of the stretching vibrations of free silanol groups (3700 cm^−1^) of the silica surface decreases. This suggests that as the polymerization of lactide proceeds, PLA simultaneously grafts to the silanol groups of the silica surface with the formation of hydrolytically unstable Si-O-C bonds. In the case of Silochrom-NH_2_ (Figure 3b), following polymerization, a characteristic band with maximum absorption at 1640 cm^−1^ can be seen, corresponding to azomethine bonds in surface compounds formed between aminopropyl carrier groups and carbonyl groups of grafted polylactide [27,28,29].

**Figure 4:**
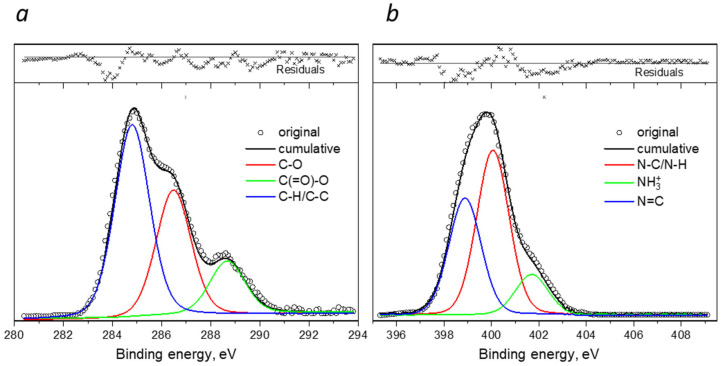
Deconvoluted XPS spectra: (**a**) C1s for silochrom/PLA and (**b**) N1s for silochrom-NH_2_/PLA.

The deconvoluted C1s (for silochrom/PLA) and N1s (silochrom-NH_2_/PLA) XPS spectra are presented in Figure 4. The C1s spectra of silochrom/PLA are represented by three carbon atoms, where C1s with binding energy E_bind_ = 284.8 eV is assigned to C-C/C-H, E_bind_ = 286.5 eV is assigned to C-O (ether bond), and E_bind_ = 288.7 eV is assigned to carboxyl carbon. These results are in line with the XPS spectra of PLA shown in recently published works [30,31]. The N1s spectra of silochrom-NH_2_/PLA confirm the results of IR analysis whereby the presence of an N atom with E_bind_ = 398.5 eV [32] indicates that polymerization of *L*-lactide on the aminated silica occurs through the formation of a C=N bond. The N1s with E_bind_ = 400.1 corresponds to the N-C bond in aminopropyl fragments, which is not bonded with *L*-lactide species. It should be noted that part of the nitrogen atoms are present in protonated form (the third peak with a maximum at 401.7 eV in Figure 4b).

**Figure 5:**
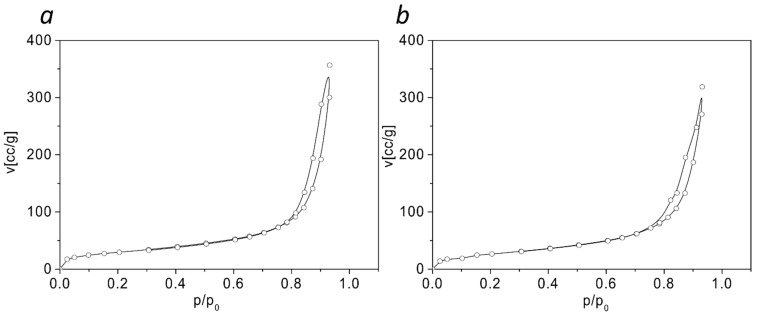
N_2_ adsorption/desorption isotherms for composites (**a**) Silochrom/PLA and (**b**) Silochrom-NH_2_ /PLA.

The porosity of samples was calculated from adsorption/desorption curves shown in Figure 5. The specific surface area, total pore volume, and values of average pore diameter of PLA/silica composite were calculated using BET and BJH methods and are presented in Table 1. It could be seen that the specific surface area, total pore volume, and average pores diameter decrease with the silica modification.

**Figure 6:**
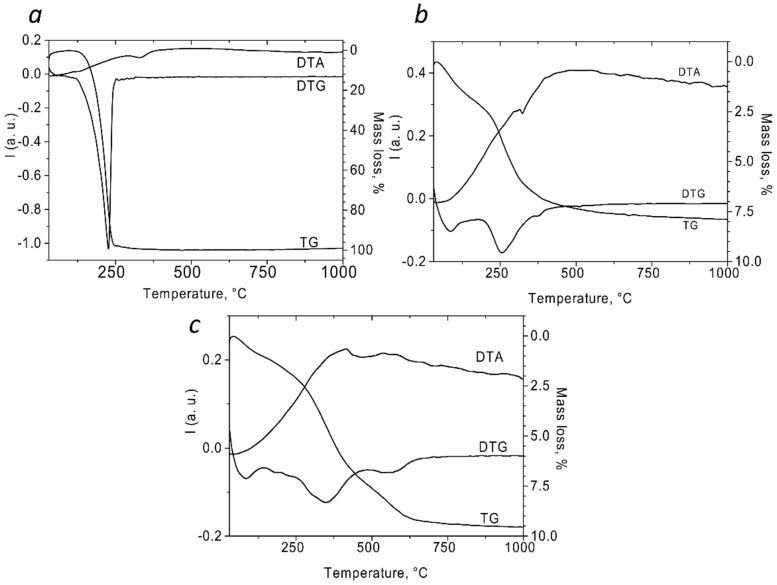
TG-, DTG-, and DTA-curves for (**a**) lactide, (**b**) Silochrom/PLA and (**c**) Silochrom-NH_2_/PLA.

TGA was carried out to investigate the influence of silica on the stability of the PLA/silica nanocomposites. DTA, TG, and DTG curves for initial lactide and synthesized composites are presented in Figure 6. According to Figure 6a, complete degradation of lactide occurred between 105 °C and 250 °C with the maximum at 225 °C. After immobilization on Silochrome and Silochrome-NH_2_, the maximum shifted to 250 °C and 270 °C, respectively. Both composites showed a 2% weight loss at a temperature of 90 °C (Figure 6b,c), corresponding to moisture evaporation. For Silochrom/PLA composite, the main decomposition process occurred in the temperature range of 200–400 °C with a maximum peak at 250 °C (Figure 6b). For Silochrom-NH_2_/PLA, two stages of thermal decomposition were detected (Figure 5c). Thermal oxidative destruction of composite begins at a temperature of 180 °C and proceeds in two stages. In the first step of degradation between 200 to 400 °C, there is initial destruction of the modifier, with a peak at 350 °C. The second decomposition step takes place at a temperature maximum of 565 (485–700 °C), where the carbonization and oxidation of decomposition products of the organic part take place. Finally, at a temperature of 700 °C, the final oxidative destruction of polylactic acid is observed. The Silochrom-NH_2_/PLA composite tended to have slightly better thermal stability in comparison with unmodified silica. This is potentially due to the stronger interactions between the PLA and the modified silica surface. The total weight loss for the Silochrom/PLA and Silochrom NH_2_/PLA composites was 7.90% and 9.52%, respectively, where 5.9% and 7.5% were attributed to the immobilized PLA on the silica and aminated silica, respectively.

### 3.2. Adsorption Studies

**Figure 7:**
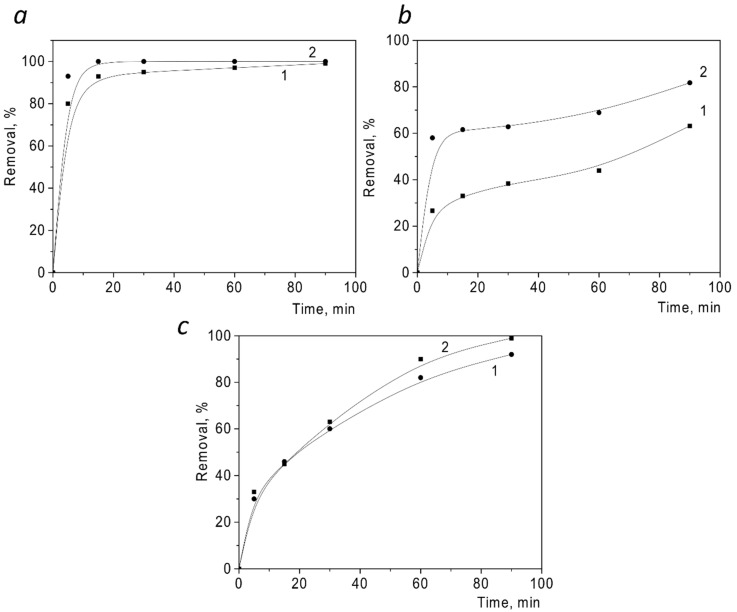
Kinetic curves of adsorption (**a**) Pb^2+^, (**b**) Co^2+,^ and (**c**) Cu^2+^—ions on Silochrom /PLA (1) and Silochrom-NH_2_/PLA (2). (Experimental conditions: mass of sorbent 0.1 g, the volume of the solution—25 mL, metal content in the solution 100 µg).

The adsorption kinetics and overall adsorption rate are significant factors for determining the efficiency of an adsorbent [33,34] and its potential use at a large scale [35,36]. Kinetics studies open the possibility of determining the mechanism of adsorption, and thus, adsorption tests that investigated the effect of contact time were performed for Pb(II), Co(II), and Cu(II) ions in aqueous solutions in the neutral media. The influence of phase contact time of metal ions adsorption using Silochrom/PLA and Silochrom-NH_2_/PLA composites are presented in Figure 7.

It can be seen from Figure 7 that the adsorption kinetics for Pb(II), Co(II), and Cu(II) ions are similar for both composites. However, Silochrom-NH_2_/PLA showed higher affinity to all selected metal ions. The highest adsorption removal was found for Cu(II) and Pb(II) ions (100%). For Pb(II), complete removal was achieved after 15 min of contact, whereas for Cu(II) ions, it took 90 min to reach 100% removal. It was found that the adsorption of Co(II) ions proceeds in two steps: an initial fast step, which occurred during the first 15 min, followed by a slower adsorption step, which occurred during the next 75 min, while the adsorbed amounts reached the equilibrium. The produced composites maintain the kinetic properties of the initial silica matrix, which is characterized by a sufficiently high rate of adsorption.

### 3.3. Effect of pH

The effect of initial pH was investigated in the pH range of 1.68 to 6.86 (Table 2). The Silochrom-NH_2_/PLA composite was found to be most effective at pH = 6.86 with ion removal of 99%, 99%, and 74% for Cu(II), Pb(II), and Co(II) ions, respectively. Adsorption of aqua complexes of selected bivalent metal ions [Co(H_2_O)_6_]^2+^, [Pb(H_2_O)_6_]^2+,^ and Cu(H_2_O)_6_]^2+^ [37] was found to be high as well and reached up to 81–99%. The low acidity of initial metal ion solutions (pH = 1–4) was found to decrease the adsorption efficiency of both composites. It can be seen from Table 2 that both hybrid materials showed similar dependency on pH. However, the Silochrom-NH_2_/PLA showed a higher degree of sorption towards the selected ions in the same pH range. Overall, in the static adsorption mode, the synthesized composites have high adsorption activity with respect to Pb(II), Co(II), and Cu(II) cations in a neutral medium with Silochrom-NH_2_/PLA showing better sorption than Silochrom/PLA composites.

### 3.4. Adsorption Isotherms

**Figure 8:**
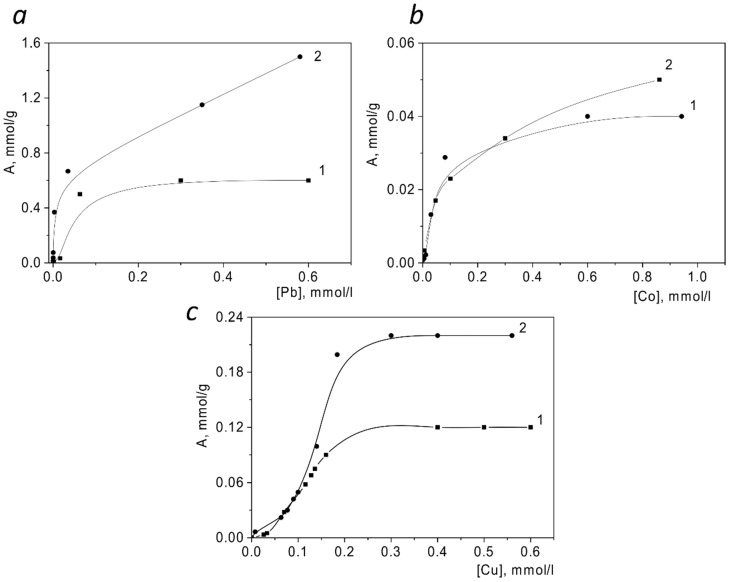
Adsorption isotherms of (**a**) Pb^2+^, (**b**) Co^2+^ and (**c**) Cu^2+^ on the surface of the synthesized composites Silochrom/PLA (1) and Silochrom-NH_2_/PLA (2).

The effect of the initial concentration of the metal ions on the adsorption behavior of silica/PLA composites was studied at room temperature and neutral pH. The initial concentrations used ranged between 2 mg∙L^−1^ and 1000 mg∙L^−1^. Typical adsorption isotherms of Pb(II), Co(II), and Cu(II) ions are shown in Figure 8, where it can be seen that both composites have a higher affinity to Pb(II) ions than to Co(II) and Cu(II) ions. The isotherms of Pb(II) and Co(II) ions have *L*-form, and Cu(II) ions are S-form, which are characterized as Langmuir and multilayer adsorption, respectively. Saturation was achieved for all bivalent metal ions studied. The adsorption capacities of the Silochrom-NH_2_/PLA were 1.5 mmol/g for Pb^2+^; 0.05 mmol/g for Co^2+^ and 0.23 mmol/g for Cu^2+^. Similarly, the adsorption capacities for the Silochrom/PLA composite were 0.50 mmol/g for Pb^2+^, 0.04 mmol/g for Co^2+,^ and 0.10 mmol/g for Cu^2+^.

Langmuir, Freundlich, and Temkin adsorption models were applied to evaluate the adsorption behavior of the composites. The characteristic parameters were calculated for each isotherm model and summarized in Table 3. According to the obtained data, all systems where metal ions are present in the form of aqua complexes (de-ionized water) fit well with the Langmuir isotherm model. The fitting was confirmed by the high values of correlation coefficients (0.8–0.99). In the systems where metal ions were adsorbed by Silochrom-NH_2_/PLA composites, adsorption behavior could be described by the Freundlich isotherm model (*R*^2^ from 0.91–0.97). This could potentially be due to a more heterogeneous surface of the Silochrom-NH_2_/PLA composite, and therefore potentially, more groups could be responsible for adsorption, resulting in multilayer adsorption with non-uniform distribution of adsorption heat.

## 4. Conclusions

Silica/PLA composites were synthesized through ring-opening polymerization of *L*-lactide onto the surface of nonmodified and modified porous silica in the presence of stannous octoate as a catalyst. The immobilization of L-lactide was confirmed by FTIR, and it was found that the thermal decomposition temperature of the composites was higher than that of the pure lactide. The obtained composites effectively removed toxic metal cations (Pb^2 +^, Co^2+,^ and Cu^2+^) from aqueous solutions. Silochrom-NH_2_/PLA composite was found to better adsorb Pb(II) Cu(II) and Co(II) ions (up to 99%, 99%, and 74%, respectively) in a neutral medium. The obtained composites, which have good kinetic properties, do not swell, have a sufficiently high adsorption capacity with respect to toxic metal cations, and are prospective sorbents for water remediation.

## Figures and Tables

**Table 1:** Structural characteristics of the composites.

Samples	Specific Surface Area, m^2^/g	Total Pore Volume, cm^3^/g	Mean Pore Diameter, nm
Silochrom	143	0.95	17
Silochrom/PLA	107	0.55	12–15
Silochrom-NH_2_/PLA	101	0.49	12–15

**Table 2:** The pH-dependent values of the maximum separation degree of Pb(II), Co(II), and Cu(II) ions by modified composites at the static mode of adsorption (Experimental conditions: mass of sorbent—0.1 g, the volume of the solution—25 mL, metal content in the solution —100 µg).

		Removal Efficiency, %			
		pH = 1.68	pH = 4.01	Distilled Water	pH = 6.86
Co(II)	Silochrom-NH_2_/PLA	0	6.3	81.7	73.9
	Silochrom/PLA	0	2.1	63.0	27.8
Pb(II)	Silochrom-NH_2_/PLA	3.4	0.7	99.3	99.7
	Silochrom/PLA	0	25.0	99.5	89.9
Cu(II)	Silochrom-NH_2_/PLA	0	64.0	86.4	99.0
	Silochrom/PLA	0	60.0	70.2	92.7

**Table 3:** The isotherm model parameters obtained for Cu(II), Co(II), and Pb(II) ions adsorption onto Silochrom/PLA and Silochrom-NH_2_/PLA composites.

Isotherm Model/ Parameter, Unit	Silochrom/PLA			Silochrom-NH_2_/PLA		
	Cu^2+^	Co^2+^	Pb^2+^	Cu^2+^	Co^2+^	Pb^2+^
Langmuir						
*q_m_*, mmol/g	0.12	0.04	0.66	0.31	0.06	1.44
*K_L_*, L/mol	7.949	70.909	17.976	5.604	8.531	49.163
R^2^	0.9356	0.9951	0.80430	5.604	0.9855	0.9775
Freundlich						
*K_F_*, mmol/g	1.59	2.59	1.12	1.96	13.89	2.55
1/*n*	1.310	1.941	0.751	0.952	0.591	0.484
R^2^	0.8757	0.8178	0.9709	0.9056	0.9665	0.9123
Temkin						
*b_T_*, kJ/mol	73.652	415.700	28.719	38.202	533.180	15.158
K_T_	47.24	629.38	1669.66	47.20	712.91	5118.5
R^2^	0.9028	0.8591	0.8304	0.7011	0.3294	0.9527

## Data Availability

Not applicable.
